# Is there a Learning Curve for Pancreaticoduodenectomy after Fellowship Training?

**DOI:** 10.1155/2010/230287

**Published:** 2011-01-24

**Authors:** Jeffrey M. Hardacre

**Affiliations:** Department of Surgery, Division of Surgical Oncology, University Hospitals Case Medical Center, 11100 Euclid Avenue, Cleveland, OH 44106-5047, USA

## Abstract

*Background*. Limited data exist regarding a learning curve for pancreaticoduodenectomy (PD). This study examines whether a learning curve exists for the performance of PD after fellowship training. *Methods*. Review of the outcomes of a single surgeon's first 60 PDs after completion of specialty training in pancreatic surgery. *Results*. Sixty PDs were performed over five years, with the final 30 being done in the last 15 months. Patient age and gender did not differ between the first 30 and last 30 patients. When comparing the first 30 PDs to the second 30 PDs, significant improvements were found in operative time (463 versus 388 minutes), length of stay (10 versus 7 days), and receipt of adjuvant therapy (58% versus 91%). There were no significant differences found in mortality (7% versus 0%), complications (60% versus 50%), readmissions (18% versus 20%), or margin-positive resections (25% versus 24%). *Conclusion*. Even with extensive training in pancreatic surgery, a learning curve exists for the performance of PD. With experience, improvements were made in operative time, but more importantly in patient outcomes including length of stay and receipt of adjuvant therapy.

## 1. Introduction

There is a growing literature about learning curves and the performance of advanced laparoscopic operations, such as fundoplication and colectomy [1, 2]. Open pancreaticoduodenectomy (PD) is a complex operation about which there are limited data regarding a learning curve. Tseng et al. examined the initial operative experiences with PD of three fellowship-trained surgeons [3]. They found that after 60 cases, each surgeon showed improvement with regard to estimated blood loss, operative time, length of stay, and achievement of margin-negative resections. They concluded that there was an “inherent learning curve” in the performance of pancreaticoduodenectomy.

A question not answered by Tseng et al. is whether fewer than 60 PDs could be a threshold beyond which improvement is seen in the performance of PD by a fellowship-trained surgeon. This study examines the outcomes of a single surgeon's first 60 PDs, assessing for changes over time.

## 2. Methods

The Institutional Review Board of University Hospitals Case Medical Center approved this study. The medical records of a single surgeon's first 60 pancreaticoduodenectomies were reviewed. During his chief resident year and an additional year of training in advanced gastrointestinal surgery, the surgeon performed 63 PDs, seven distal pancreatectomies, and five total pancreatectomies. The operations examined in this study occurred over five years (July 2004–June 2009), with the final 30 cases done over the last 15 months. 

The data were analyzed comparing the first 30 cases (Group 1) to the last 30 cases (Group 2). Overall complications, including pancreatic fistula and delayed gastric emptying (DGE), were scored using recognized classification systems [4–6]. The pancreaticojejunostomy was performed using an invaginating technique [7]. Postoperative care followed a pathway similar to that described by Kennedy et al. [8]. Continuous variables were analyzed by the Wilcoxon rank sum method and categorical variables by the chi-square test or Fisher's exact test, where appropriate. 

## 3. Results

The median (interquartile range, IQR) ages of the patients in groups 1 and 2 were 69 (63–76) and 65 (56–73) years, *P* = .13. Forty-seven and 50% of patients in groups 1 and 2, respectively, were female, *P* = .80.

All operations were performed with the assistance of residents or fellows. The postgraduate year (PGY) differed between groups 1 and 2. In group 1, 3%, 43%, 47%, and 7% of the cases were done with PGY3, PGY4, PGY 5, and PGY6 help. In group 2, the respective percentages were 0%, 80%, 13%, and 7%, *P* = .006.

Forty-three percent of patients in group 1 underwent a pylorus-preserving PD whereas 50% underwent a pylorus-preserving PD in group 2, *P* = .61. Median (IQR) estimated blood loss did not differ between groups, 778 (500–1250) ml versus 800 (450–1025) ml, *P* = .75. Median (IQR) operative time did decrease from group 1 to group 2, 463 (412–540) minutes versus 388 (338–420) minutes, *P* = .002. An equal percentage of patients in both groups had soft glands, 43% versus 45%, *P* = .99.

Morbidity and mortality are shown in Table 1. Two patients died in group 1 and none in group 2, but this difference was not statistically significant. The first patient died on postoperative day 24 from complications associated with bleeding that could not be controlled angiographically and required a return to the operating room. The second patient died on postoperative day 20 during a percutaneous endoscopic gastrostomy being done for refractory DGE. There were no differences in morbidity, and in particular pancreatic fistula, DGE, or wound infection, between groups 1 and 2.

Median (IQR) length of stay decreased from group 1 to group 2, 10 (8–17) versus 7 (6–11) days, *P* = .02. Rates of reoperation, 20% versus 10% (*P* = .47) and readmission, 18% versus 20% (*P* = .99) did not differ between groups. Reasons for reoperation are shown in Table 2.

Final pathology, tumor size, and R1 resection status did not differ between groups, Table 3. For patients with periampullary adenocarcinoma, a greater percentage of patients in group 2 received adjuvant therapy, 58% versus 91%, *P* = .02. A separate, similar analysis (data not shown) comparing the first 20 cases to the last 20 cases revealed similar, significant differences in operative time, length of stay, and receipt of adjuvant therapy.

## 4. Discussion

Learning curves have been described for a number of advanced, minimally invasive operations [1, 2]. Limited data exist, however, regarding the learning curve for PD, one of the most complex operations performed by surgeons. Tseng et al. analyzed the learning curve for PD by looking at the initial operative experience of three, fellowship-trained surgeons [3]. Their analysis found that after 60 cases, significant improvements were achieved in estimated blood loss (1100 versus 725 ml), operative time (589 versus 513 minutes), length of stay (15 versus 13 days), and R1 resection rate (30% versus 8%). They did not analyze other outcomes such as mortality, morbidity, rates of reoperation/readmission, or receipt of adjuvant therapy. They concluded that PD has an “inherent learning curve,” and that after 60 cases improvements were seen in the metrics noted above.

Data from the current study confirm the findings of Tseng et al. with regard to the presence of a learning curve for PD after fellowship training. Similar to Tseng et al., this study showed improvements in operative time and length of stay with experience. However, in this study, improvements were observed after 30 cases. Certainly, neither the data from this study nor the study of Tseng et al. define the learning curve for PD after fellowship training. They simply demonstrate that one exists. What experience constitutes the learning curve may depend on a number of factors including individual ability as well as training volume in the preoperative, operative, and postoperative care of patients requiring pancreatic surgery. The preoperative and postoperative care components of patients undergoing PD can not be underestimated as knowing whom to operate on and how to manage complications may be just as important to patient outcomes as is the technical performance of the operation.

The concept of a learning curve correlates with the abundant literature on volume-outcome relationships, particularly those described for PD [9–11]. Those data show that individual surgeon volume as well as hospital volume have a direct impact not only on short-term patient outcomes, but also on long-term outcomes, such as survival. The concept of the volume-outcome relationship and the presence of a learning curve for PD *after* fellowship training are especially important given that, according to the Accreditation Council for Graduate Medical Education, graduating US surgical residents in 2009 performed an average (standard deviation) of 5.6 (5) pancreaticoduodenectomies during their training. These data clearly show that the average graduating surgical resident in the U.S. who is not fellowship trained is very early in his or her learning curve for mastering PD. Further, such data in the future may influence hospital credentialing, as, in general, documentation beyond general surgical training is almost always needed for advanced laparoscopic procedures, but not for complex open procedures such as PD, esophagectomy, or hepatectomy.

Other findings of this study that warrant discussion include the changing nature of the level of resident involved in the operation. Part of the way through the Group 1 experience, our surgical services were rearranged such that the senior resident on the author's service changed from a PGY5 to a PGY4. Such a change did influence how much of an operation the resident performed. Uniformly, the resident performed the biliary and gastrointestinal anastomoses. However, whether the resident performed the pancreaticojejunostomy depended on their year of training, gland texture, and individual ability. Perhaps the most intriguing finding of the study, though, was that patients were more likely to receive adjuvant therapy after resection for periampullary adenocarcinoma in group 2 (91%) versus Group 1 (58%). This occurred despite no significant changes in complications, reoperations, or readmissions. This outcome is of importance as adjuvant therapy after resection for pancreatic cancer has been shown to increase survival, but single-institution studies show that as few as 47% to 74% of patients receive adjuvant therapy [12, 13]. Explanations for the increase in adjuvant therapy may include a 10% decrease, though not significant, in Clavien 3-4 complications as well as active involvement by the author in the discussion and planning of adjuvant therapy in the later phases of the study period. During that time, the author helped introduce an adjuvant therapy trial at the institution.

The most obvious limitation to this study is that it examines a single surgeon's initial operative experience and that may not be generalizable to all fellowship trained surgeons. Also, the number of patients studied is somewhat small such that some clinically relevant differences between groups 1 and 2 may not have achieved a statistically significant level. The current data do approximate the outcomes of Tseng et al. as well as the outcomes of a large, single-institution experience with PD [14]. Finally, what constitutes a fellowship-trained surgeon? There are numerous pathways to achieve further training in pancreatic surgery: surgical oncology, transplantation, and hepatopancreaticobiliary surgery. The experience across fellowship disciplines and across institutions certainly differs.

PD is a complex operation with an associated learning curve. The data presented in this study corroborate the existing literature that even after fellowship training, improvements in operative and patient outcomes are achieved with experience. The learning curve for an individual surgeon is likely to vary.

## Figures and Tables

**Table 1:** Complications.

	Group 1	Group 2	*P*-value
Mortality	7%	0%	.50
Morbidity	60%	50%	.44
Clavien 1-2	27%	33%	
Clavien 3-4	27%	17%	
DGE	23%	17%	.75
A	10%	10%	
B	3%	7%	
C	10%	0%	
Fistula	30%	13%	.21
A	7%	0%	
B	17%	13%	
C	7%	0%	
Wound infection	13%	23%	.51

**Table 2:** Reasons for reoperation.

Group 1
Percutaneous endoscopic gastrostomy for DGE (POD 20)
Laparotomy for bleeding (POD 24)
Fascial debridement/closure with biologic mesh (POD 8)
Conversion loop gastrojejunostomy to Roux-en-Y gastrojejunostomy for DGE (1.5 months)
Fascial debridement/closure with biologic mesh (POD 11)
Gastrectomy for gastric staple line fistula (2.5 months)

Group 2

Lysis of adhesions for small bowel obstruction (1.5 months)
Subcutaneous debridement/vacuum dressing placement (POD 24)
Subcutaneous debridement/vacuum dressing placement (4 months)

POD: postoperative day.

**Table 3:** Pathology.

	Group 1	Group 2	*P*-value
Malignant	93%	77%	0.15
*Tumor Size (cm)	3.5 (2.5–4)	3.0 (2.5–4)	0.46
*R1	25%	24%	0.99

Tumor size reported as median (interquartile range).

*Tumor size and R1 are for pancreatic adenocarcinoma.
